# Propagation Measurements for IQRF Network in an Urban Environment

**DOI:** 10.3390/s22187012

**Published:** 2022-09-16

**Authors:** Mohammed Bouzidi, Marshed Mohamed, Yaser Dalveren, Arild Moldsvor, Faouzi Alaya Cheikh, Mohammad Derawi

**Affiliations:** 1 Department of Electronic Systems, Norwegian University of Science and Technology, 2821 Gjøvik, Norway; 2 Department of Manufacturing and Civil Engineering, Norwegian University of Science and Technology, 2821 Gjøvik, Norway; 3 Department of Avionics, Atilim University, Incek Golbasi, Ankara 06830, Turkey; 4 Department of Computer Science, Norwegian University of Science and Technology, 2821 Gjøvik, Norway

**Keywords:** IQRF, path loss, wireless sensor networks, propagation, channel modelling

## Abstract

Recently, IQRF has emerged as a promising technology for the Internet of Things (IoT), owing to its ability to support short- and medium-range low-power communications. However, real world deployment of IQRF-based wireless sensor networks (WSNs) requires accurate path loss modelling to estimate network coverage and other performances. In the existing literature, extensive research on propagation modelling for IQRF network deployment in urban environments has not been provided yet. Therefore, this study proposes an empirical path loss model for the deployment of IQRF networks in a peer-to-peer configured system where the IQRF sensor nodes operate in the 868 MHz band. For this purpose, extensive measurement campaigns are conducted outdoor in an urban environment for Line-of-Sight (LoS) and Non-Line-of-Sight (NLoS) links. Furthermore, in order to evaluate the prediction accuracy of well-known empirical path loss models for urban environments, the measurements are compared with the predicted path loss values. The results show that the COST-231 Walfisch–Ikegami model has higher prediction accuracy and can be used for IQRF network planning in LoS links, while the COST-231 Hata model has better accuracy in NLoS links. On the other hand, the effects of antennas on the performance of IQRF transceivers (TRs) for LoS and NLoS links are also scrutinized. The use of IQRF TRs with a Straight-Line Dipole Antenna (SLDA) antenna is found to offer more stable results when compared to IQRF (TRs) with Meander Line Antenna (MLA) antenna. Therefore, it is believed that the findings presented in this article could offer useful insights for researchers interested in the development of IoT-based smart city applications.

## 1. Introduction

The Internet of Things (IoT) concept is expected to fulfil demands in the use of smart systems. Mainly, IoT is a network of interconnected devices equipped with tiny sensors. As communication between the devices as well as the users is enabled with the help of the Internet, it becomes possible to develop several intelligent applications for healthcare, automotive, and many other industries. Thus, it enables people to utilize such applications in order to facilitate the activities of daily life. However, the development of IoT technology presents a challenging issue due to the diversity of these applications. Particularly, when a growing interest in building IoT-based smart cities is regarded, it is necessary to consider common requirements of IoT devices such as low cost, low power consumption, and wide coverage for typical smart city applications [1]. In order to satisfy these requirements, several communication technologies such as 6LoWPAN [2], Wi-Fi [3,4], BLE [5], and ZigBee [6,7] have already been developed. However, the main concern of these technologies is the limitation of network coverage. For this reason, they are commonly preferred for indoor applications. To improve network coverage, Low-Power Wide Area Network (LPWAN) technologies such as SigFox, NB-IoT, Ingenu, and LoRa have been designed and implemented in the commercial market [8]. Alternatively, IQRF is a promising technology that provides efficient solutions in the field of IoT. Hence, as discussed in [9] several studies considering the use of IQRF in IoT applications have been recently presented in the literature [10,11,12,13,14,15,16,17,18,19].

A network based on IQRF in urban areas requires an accurate prediction of network coverage. Therefore, in order to design such networks, any propagation impairments affecting the propagation links using actual IQRF transceivers (TRs) need to be analyzed comprehensively. In this context, the most significant impairment could be assigned to path loss. Obviously, it provides useful information about variations in the received signal power with respect to the distance between transceivers, link budget, and the corresponding signal-to-noise ratio. Theoretically, path loss is highly affected by the environmental conditions. Thus, it is evident that the real-world deployment of IQRF networks requires efficient path loss modelling. Moreover, the characteristics of IQRF links in urban environments have thus far not been thoroughly studied in the existing literature. More specifically, empirical path loss models have not been proposed for the deployment of IQRF wireless networks. In addition, most works that have centered on the characteristics of wireless links in urban environments, have used a transmitter or base station at high elevation, and a receiver or mobile station at lower elevation in the measurements. However, these studies do not apply to IQRF applications in which a mesh network in an urban environment can be formed between sensors at a similar height from the ground.

In the related works, very few studies that deal with the propagation aspects of IQRF networks have been presented in [20,21,22]. In [20], real measurements and simulations of the Received Signal Strength Indicator (RSSI) are performed in three main scenarios: indoor, open-space outdoor and urban-area outdoor. In the same work, the simulated RSSI values are compared with the real performed measurements in order to demonstrate the IQRF network range perfomance. Similarly, in [21], RSSI values of an IQRF network with nodes placed at different locations of a multi-floor building (indoor) are measured. However, no changes in the distance are conducted. Rather, only the transmission power is set to different levels. The obtained results demonstrate to what extent the IQRF network is stable when the maximum transmission power is used. Another study in [22], presents the possibilities for deploying a low-power mesh network in an indoor environment using IQRF technology. In this work, RSSI is also measured for three receiving nodes and compared with the sensitivity level that each receiving node allows. Therefore, motivated from the aforementioned discussions, it is evident that there has been no published work on propagation modelling for the deployment of IQRF networks in urban environments.

Major contributions of this article include preliminary propagation measurements to be used in the construction of an IQRF network in a urban environment. Moreover, the aim is to evaluate the performance of empirical path loss models in a low-height Peer-to-Peer (P2P) configured system. To this end, firstly, measurement campaigns are performed in an urban environment by utilizing two types of IQRF transceivers operating in the 868 MHz band. In the measurements, IQRF transceivers are intended to be used as IoT devices that could be connected either at the same street in Line-of-Sight (LoS) link, two perpendicular streets in Non-Line-of-Sight (NLoS) with one single knife-edge link (one turn), or two parallel streets in NLoS with double knife-edge link (two turns). Then, the measurement results are compared with well-known empirical path loss models. Notably, although there are an abundance of measurement-based models to statistically characterize the wireless channels, our purpose is to show that which well-known empirical path loss models can be used in the construction of IQRF networks in urban environments. According to the comparison results achieved from various measurement scenarios, the empirical path loss models with higher prediction accuracy are proposed for usage in the efficient deployment of IQRF networks in an urban environment. To the best of our knowledge, no such work in the existing literature has put forth such a claim.

The structure of this article is organized as follows. In Section 2, an overview of IQRF technology is presented. Section 3 presents well-known empirical path loss models for the deployment of Wireless Sensor Networks (WSNs) in urban environments. The field measurements along with the experimental setup are described in Section 4. Next, measurement results and data analysis are provided in Section 5. Finally, concluding remarks are provided in Section 6.

## 2. Overview of IQRF Network

IQRF technology, which has recently been developed for wireless connectivity, offers low-cost wireless solutions for smart cities as comprehensively discussed in [9]. For the sake of clarity, before presenting the field measurements, an overview of IQRF technology and its potential and practical usage in smart city applications is presented in this section.

### 2.1. A Brief Introduction to IQRF Technology

IQRF is a complete technology/platform from the general perspective of the IoT concept. It includes transceivers, gateways, development tools, protocols, and supporting services. It provides reliable, low-power, low-speed, and low-data wireless connectivity in sub-GHz Industrial, Scientific and Medical (ISM) bands (433 MHz, 868 MHz, and 916 MHz). The coverage range extends from tens to hundreds of meters and more, reaching up to several kilometers in certain conditions. Thus, it can generally be employed for different wireless application domains. This technology is based on packet-oriented communication. Additionally, using IQRF technology is simple and ideal for the implementation of IoT.

Initially, IQRF technology was developed for building automation systems [20]. However, over the last decade, development of this technology has noticeably shifted towards becoming a competitive alternative to other similar technologies in this area [9]. Accordingly, use-case studies have been developed in the literature for telemetry [23], automated meter reading/smart meters [13,24,25], wireless sensor networks [26], smart homes [27], and many other domains. On the other hand, compared to other similar commercial solutions, case studies utilizing IQRF technology in the field of smart city solutions are considerably limited [10,28]. Therefore, this study also aims to increase awareness among researchers about this technology. The following sub-sections are devoted to the presentation of some technical details concerning IQRF technology.

### 2.2. Designing IoT Applications with IQRF

**Figure 1:**
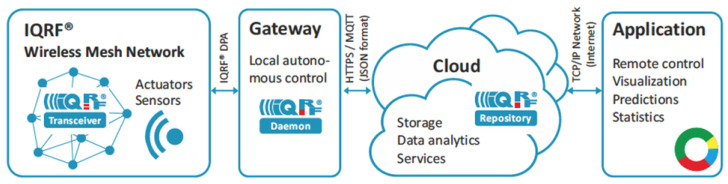
Typical IoT Application with IQRF network Design [29].

In order to provide a complete and effective IoT solution, a typical design using IQRF is depicted in Figure 1. As an IoT solution, IQRF consists of a network, gateway, cloud, and end-user applications. These components are briefly described in the following figure.

#### 2.2.1. IQRF Mesh Network

The IQRF Alliance has developed its own mesh networking protocol called IQMESH. With this protocol, data packets are delivered through a smart-routing mechanism which may overcome some the drawbacks of Star topology in terms of robustness, reliability, range and security. The building blocks of each IQRF network are its IQRF transceiver (TR) modules. These TR modules are tiny boards that include a microcontroller unit (MCU) and an RF circuitry. A built-in operating system is also developed and implemented into the MCU. Its main task is to organize the transceiver’s different operations such as Radio Frequency (RF) communication functions and serial communication with the computer for programming and debugging.

In an IQRF network, at least two application approaches are implemented. The first approach is based on the user application layer under the Operating System (OS). Basically, the OS uses predefined OS functions. In this approach, the programming is based on C language. However, this approach is not supported by IQMESH protocol. For this reason, it can be considered as a lower level programming approach destined to engineers and developers. The second approach is a higher level approach based on three-layer IQRF architecture including Hardware Profile (HWP). In fact, HWP implements the byte-oriented protocol called Device Peripheral Access (DPA) above the OS for simpler network implementation and management. Thus, programming is not required at this level, and functionality is achieved through simple control commands. However, programming is possible and optional by means of a custom DPA handler in C language. This is accomplished by extending the HWP through programming. It is worth noting that this presents a unique approach for RF communication transceivers worldwide. Moreover, two RF communication modes can be implemented in an IQRF network: (a) non-networking, (b) networking. In non-networking mode, networking functions are not supported. Hence, this mode is suitable for two or more P2P devices. Within the maximum achievable range, the packets are accessible for all devices. However, in the networking mode, there is one node that acts as a coordinator that manages a mesh network containing up to 239 nodes for a single network. In such a mode, only the addressed nodes can access the data packets sent. For more devices in the network, every node can serve as a sub-coordinator. Thus, the number of nodes can increase to up to 65,000 devices in a single network. In addition, every node is able to route in the background making it possible for a packet to hop up to 240 times to reach the destination. Packet routes can be found and created automatically as a virtual routing structure with the help of a discovery tool.

#### 2.2.2. IQRF Gateway

Nodes in an IQRF architecture can be directly controlled by a cloud server. To achieve this, simply, a gateway is required. For this reason, the IQRF Alliance has developed hardware specific gateways such as Wi-Fi, GSM and Ethernet gateways to provide connections between IQRF and the Internet. Additionally, these gateways include a user-programmable sensor node that is usually programmed to act as a network coordinator. Moreover, IQRF Daemon, which is a Linux-based and open-source software specific gateway, is also developed. The IQRF gateway can then be implemented in Linux-based single-board computers such as Raspberry Pi [30]. In addition, an IQRF Software Development Kit (SDK) is also provided to support devices that do not support any OS such as standalone MCUs.

#### 2.2.3. IQRF Cloud Server

The IQRF Cloud provides an effective way to exchange data between IQRF wireless device(s) and a user super-ordinary system implemented by a higher level platform such as PHP, JavaScript or web interfaces. To achieve this, the IQRF Cloud operates with IQRF gateways which provides Internet connectivity via Ethernet, GPRS or WiFi. Owing to the IQRF Cloud requiring minimal configurations, the IQRF Cloud enables easy data access, processing and visualization. Additionally, IQRF networks can be connected to any other cloud. Usually, this is accomplished by the IQRF gateway software Daemon running on a Linux-based single-board computer. The typical communication interface provided by Daemon gateway is standard MQTT with JSON.

## 3. Empirical Path Loss Models

In this section, well-known empirical path loss models for urban links are briefly described.

### 3.1. Log-Distance Model

The general form for an empirical path loss log-distance model is defined as follows;

(1) $${PL_{\mathrm{LG}}\left(d\right)}_{\left[\mathrm{dB}\right]}={L(d_{0})}_{\left[\mathrm{dB}\right]}+10n\log_{10}\left(\frac{d}{d_{0}}\right)$$

where *n* is the path loss exponent, $L(d_{0})$ is the path loss in dB at a far-field distance or reference distance $d_{0}$ in meters. *d* is the transmitter–receiver (Tx–Rx) separation distance in meters. Due to the multipath effects, the path loss for the same Tx to Rx distance might be different. By considering these effects, Equation (1) becomes

(2) $${PL_{LG}\left(d\right)}_{\left[\mathrm{dB}\right]}={L(d_{0})}_{\left[\mathrm{dB}\right]}+10n\log_{10}\left(\frac{d}{d_{0}}\right)+X_{\sigma }$$

where $X_{\sigma }$ is a zero mean Gaussian random variable with standard deviation σ when expressed in the dB scale and describes the shadowing effects [31]. Parameters $L(d_{0})$ and *n* can be estimated by performing linear regression with the measurement data, while σ(dB) may be determined from experimental data using Equation (3)

(3) $$\sigma_{\left[dB\right]}=\sqrt{\sum_{i=1}^{N}\frac{{\left(L_{meas}\left(i\right)-L_{pred}\left(i\right)\right)}^{2}}{N}}$$

where $L_{meas}\left(i\right)$ and $L_{pred}\left(i\right)$ are the measured and predicted average path loss at point *i*, respectively, and *N* is the total number of path loss samples [32].

### 3.2. Free-Space Model

The Free-Space Path Loss (FSPL) model is the lower bound estimation of link losses. This model assumes that the transmit antenna and receive antenna are located in an open environment with no absorbing objects or reflecting surfaces. This model is obtained utilizing the well-known FSPL model given by [33]

(4) $$L_{\mathrm{FSPL}}{\left(D,F\right)}_{\left[dB\right]}=32.44+20\log\left(D\right)+20\log\left(F\right)$$

where *D* is the Tx–Rx separation distance in km and *F* is the frequency in MHz.

### 3.3. COST231 Walfisch–Ikegami (COST231-WI) Model

The COST231-WI model is considered as one of the most efficient propagation models for urban and suburban environments with more or less regular positioning of buildings [34]. This model relies on the Walfisch–Bertoni model [35] and the Ikegami model [36] to calculate the multiple screen forward diffraction loss for high antennas. In the case of low-height antennas, however, it is measurement-based. The other terms of this model are the free-space term, losses due to diffraction caused by interactions with street objects, and the factor of street orientation. Moreover, due to its higher prediction accuracy, it has become one of the most popular path loss models in recent years [37,38,39].

For the LoS condition, the equation of this model can be written as

(5) $$PL_{\mathrm{LoS}}{\left(D,F\right)}_{\left[\mathrm{dB}\right]}=42.6+26\log\left(D\right)+20\log\left(F\right),\;\;\mathrm{for}\;D⩾0.02\;\mathrm{km}.$$

For the NLoS condition, the model is written as:

(6) $$PL_{\mathrm{NLoS}}=\left\{\begin{array}{cc} L_{\mathrm{FSPL}} & \mathrm{when}\;L_{\mathrm{rts}}+L_{\mathrm{msd}}<0\\ L_{\mathrm{FSPL}}+L_{\mathrm{rts}}+L_{\mathrm{msd}}\; & \mathrm{otherwise} \end{array}\right.$$

where $L_{\mathrm{FSPL}}$ denotes the free-space losses defined in (4), $L_{\mathrm{rts}}$ is the roof top to street diffraction, and $L_{\mathrm{msd}}$ represents multi-screen diffraction losses. We put $\Delta h_{\mathrm{Tx}}=h_{\mathrm{Tx}}-h_{\mathrm{roof}}$ and $\Delta h_{\mathrm{Rx}}=h_{\mathrm{roof}}-h_{\mathrm{Rx}}$, where, $h_{\mathrm{Tx}}$ and $h_{\mathrm{Rx}}$ are the Tx and Rx antenna heights above the ground in meters, respectively. The roof top to street diffraction is calculated as:

(7) $$L_{\mathrm{rts}}=\left\{\begin{array}{cc} \begin{array}{c} -16.9-10\log_{10}\left(w\right)+10\log_{10}\left(F\right)\\ +20\log_{10}\left(\Delta h_{\mathrm{Rx}}\right)+L_{\mathrm{ori}} \end{array}\; & \mathrm{for}\;h_{\mathrm{roof}}>h_{\mathrm{Rx}}\\ 0 & \mathrm{for}\;L_{\mathrm{rts}}<0 \end{array}\right.$$

where

(8) $$L_{\mathrm{ori}}=\left\{\begin{array}{cc} -10+0.354\varphi & \mathrm{for}\;0^{\circ }⩽\varphi ⩽35^{\circ }\\ 2.5+0.075(\varphi -35) & \mathrm{for}\;35^{\circ }⩽\varphi ⩽55^{\circ }\\ 4-0.114(\varphi -55) & \mathrm{for}\;55^{\circ }⩽\varphi ⩽90^{\circ } \end{array}\right.$$

The multi-screen diffraction loss $L_{\mathrm{msd}}$ is calculated as:

(9) $$L_{\mathrm{msd}}=\left\{\begin{array}{cc} L_{\mathrm{bsh}}+K_{a}+K_{d}\log_{10}\left(D\right)+ & \\ K_{f}\log_{10}\left(F\right)-9\log_{10}\left(b\right) & \\ 0 & \;\mathrm{if}\;L_{\mathrm{msd}}<0 \end{array}\right.$$

where

(10) $$L_{\mathrm{bsh}}=\left\{\begin{array}{cc} -18\log_{10}\left(1+\Delta h_{\mathrm{Tx}}\right) & \mathrm{for}\;h_{\mathrm{Tx}}>h_{\mathrm{roof}}\\ 0 & \mathrm{for}\;h_{\;\mathrm{Tx}}⩽h_{\mathrm{roof}} \end{array}\right.$$

*b* is the building-to-building distance in meters, φ is the street orientation angle in degrees, and *w* is the street width in meters. Parameters $k_{a}$, $k_{d}$ and $k_{f}$ are calculated as follows:

(11) $$k_{a}=\left\{\begin{array}{cc} 54-0.8\Delta h_{\mathrm{Tx}} & \mathrm{for}\;D⩾0.5\;\mathrm{km}\;\mathrm{and}\;h_{\mathrm{Tx}}⩽h_{\mathrm{roof}}\\ 54-0.8\Delta h_{\mathrm{Tx}}\left(\frac{D}{0.5}\right) & \mathrm{for}\;D<0.5\;\mathrm{km}\;\mathrm{and}\;h_{\mathrm{Tx}}⩽h_{\mathrm{roof}}\\ 54 & \mathrm{for}\;h_{\mathrm{Tx}}>h_{\mathrm{roof}} \end{array}\right.$$

(12) $$k_{d}=\left\{\begin{array}{cc} 18-15\left(\frac{\Delta h_{\mathrm{Tx}}}{h_{\mathrm{roof}}}\right) & \mathrm{for}\;h_{\mathrm{Tx}}⩽h_{\mathrm{roof}}\\ 18 & \mathrm{for}\;h_{\mathrm{Tx}}>h_{\mathrm{roof}} \end{array}\right.$$

(13) $$k_{f}=\left\{\begin{array}{cc} -4+1.5\left(\frac{F}{925}-1\right) & \mathrm{for}\;\mathrm{urban}\;\mathrm{areas}\\ -4+0.7\left(\frac{F}{925}-1\right) & \mathrm{for}\;\mathrm{suburban}\;\mathrm{areas} \end{array}\right.$$

### 3.4. Okumura–Hata Model

The Okumura–Hata or simply the Hata model is a widely used empirical propagation model for predicting transmission losses in urban, suburban and rural (open area) locations. In this model, parameters such as reflections, scattering, and diffraction caused by surrounding structures are taken into account. Moreover, four metric and physical parameters, namely Rx–Tx distance, Tx antenna height, Rx antenna height and the carrier frequency, are involved in Hata path loss estimation. The path loss of this model is calculated as follows [40]:

(14) $$PL{\left(D,F\right)}_{\left[\mathrm{dB}\right]}=A+B+C$$

where

(15) $$A=69.55+26.16\log_{10}\left(F\right)-13.82\log_{10}\left(h_{\mathrm{Tx}}\right)-a\left(h_{\mathrm{Rx}}\right)$$

(16) $$B=\left(44.9-6.55\log_{10}\left(h_{\mathrm{Tx}}\right)\right)\log_{10}\left(D\right)$$

(17) $$C=\left\{\begin{array}{cc} 0, & \mathrm{for}\;\mathrm{urban}\;\mathrm{areas}\\ -2{\left(\log_{10}\left(\left(F\right)/28\right)\right)}^{2}-5.4, & \mathrm{for}\;\mathrm{suburban}\;\mathrm{areas}\\ \begin{array}{c} -4.78{\left(\log_{10}\left(F\right)\right)}^{2}+18.33\\ \log_{10}\left(F\right)-40.94, \end{array}\; & \mathrm{for}\;\mathrm{rural}\;\mathrm{areas} \end{array}\right.$$

and, $a(h_{\mathrm{Rx}})$ is the Rx antenna height gain correction factor that depends on the environment and is calculated as:

(18) $$a\left(h_{\mathrm{Rx}}\right)=\left(1.1\log_{10}\left(F\right)-0.7\right)h_{\mathrm{Rx}}-\left(1.56\log_{10}\left(F\right)-0.8\right)$$

### 3.5. COST231-Hata Model

This model is an extended version of the Okumura–Hata model. It is widely used to predict path loss in mobile communication. Moreover, this model contains adjustments for urban, suburban, and rural environments. In this model, path loss is computed as [41]:

(19) $$\begin{array}{c} PL{\left(D,F\right)}_{\left[\mathrm{dB}\right]}=46.3+39.9\log\left(F\right)-13.82\log\left(h_{\mathrm{Tx}}\right)\\ -a\left(h_{\mathrm{Rx}}\right)+\left(44.9-6.55\log_{10}\left(h_{\mathrm{Tx}}\right)\right)\log_{10}\left(D\right)+C_{m} \end{array}$$

where $C_{m}$ is defined as 0 dB for a suburban or rural environment, while it is defined as 3 dB for an urban environment. The function $a(h_{\mathrm{Rx}})$ is defined for an urban environment as

(20) $$a\left(h_{\mathrm{Rx}}\right)=3.20{\left(\log_{10}\left(11.75h_{\mathrm{Rx}}\right)\right)}^{2}-4.97,\;\mathrm{for}\;F>400\;\mathrm{MHz}$$

However, for a suburban or rural environment, $a(h_{\mathrm{Rx}})$ is the same as (18).

Overall, the above-mentioned path loss models are comparatively assessed in Table 1 to summarize their strengths and weaknesses.

## 4. Measurement Campaigns

Propagation measurements are performed to assess the accuracy of the well-known empirical path loss models for a specific deployment of the IQRF network in an urban environment. In the following sections, first, measurement scenarios are presented, and then the measurement setup is described.

### 4.1. Measurement Scenarios

**Figure 2:**
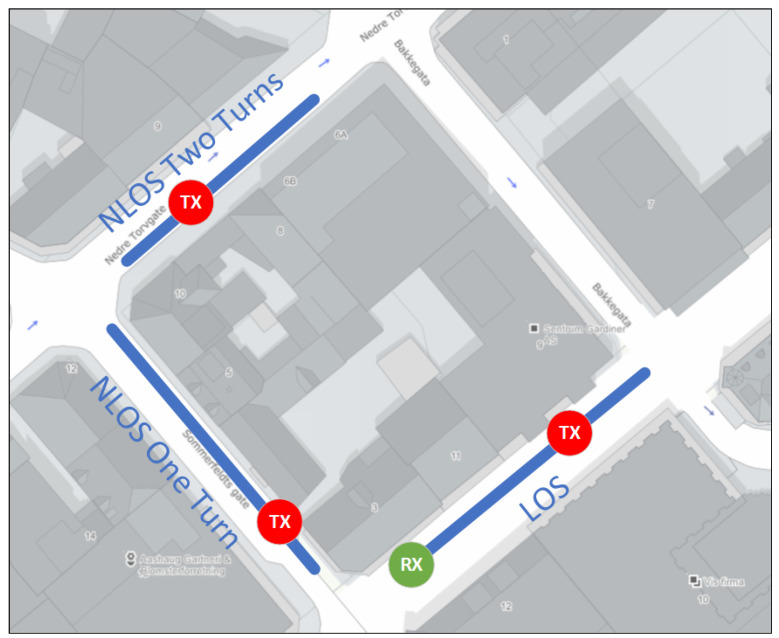
Satellite view of the area in which the measurements were conducted, the Rx was located at one location while the Tx was placed at different locations.

WSNs are often deployed to monitor and act on the physical world. However, nodes in WSNs are not always distributed uniformly. For instance, in an urban environment, streets are commonly formed by locally rectangular or pseudo-rectangular buildings. Hence, in such an environment, WSNs can be implemented in many different ways along the streets. In this study, we consider three different scenarios that lead automatically to three different radio links. The considered scenarios are depicted in Figure 2. The details of the scenarios are described in the following subsections.

#### 4.1.1. Line-of-Sight (LoS) Link

**Figure 3:**
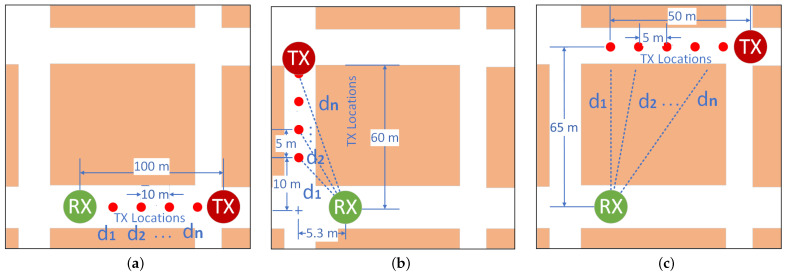
Measurement scenarios. (**a**) Line-of-Sight scenario, (**b**) Non-Line-of-Sight scenario (one turn), (**c**) Non-Line-of-Sight scenario (two turns).

This link arises when the Tx and Rx are on the same straight section of the street. In an LoS scenario, the distance between two consecutive Tx locations was chosen to be 10 m. Locations, distances, and the corresponding values used for this scenario are illustrated in Figure 3a. It is worth mentioning that although TRs are on the same street (LoS), there are still obstacles in between such as cars, cafes, chairs, people, and so forth.

#### 4.1.2. Non-Line-of-Sight (NLoS): One-Turn Link

This link arises when the Tx and Rx are located in two streets that intersect each other. In this link, the radio signal propagates in the space from the Tx to reach the Rx, after turning one corner. In this scenario, the distance between two consecutive Tx locations was chosen to be 5 m, as the signal fades faster compared to the LoS link. Additionally, $d_{i}$ represents the shortest distance in meters, which was calculated using the Pythagoras theorem, separating the Tx from the Rx. Locations, distances, and the corresponding values used for this scenario are illustrated in Figure 3b.

#### 4.1.3. Non-Line-of-Sight (NLoS): Two-Turns Link

This link arises when the Tx and Rx are placed in two parallel streets. In this link, the radio signal propagating from the transmitter to receiver is expected to turn two corners. The distance between two consecutive Tx locations was also chosen to be 5 m in this scenario. Similar to the previous scenario, the distances $d_{i}$ were calculated using the Pythagoras theorem. Locations, distances, and the corresponding values used for this scenario are illustrated in Figure 3c.

### 4.2. Measurement Setup

**Figure 4:**
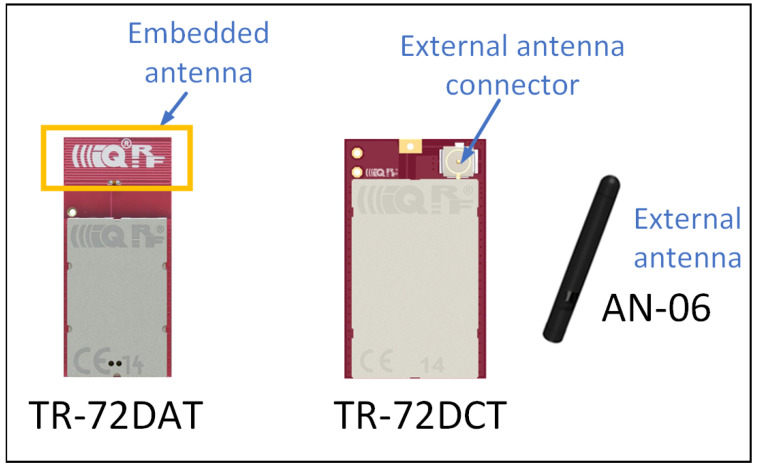
IQRF transceivers used in the measurement.

In the measurements, two types of IQRF TRs are used. One of the types contains a Meander Line Antenna (MLA) etched on the Printed Circuit Board (PCB) of the TR itself. It does not require any external components. In this paper, this type of TR is referred to as a transceiver with embedded antenna. On the other side, the other type of TR has a connector in order to connect an external Straight-Line Dipole Antenna (SLDA). In the following sections of this paper, this type of TR is referred to as a transceiver with external antenna. Both types of TRs are depicted in Figure 4.

In the measurements, one of the TRs is configured as a transmitter (Tx) transmitting packets continuously. The other one is configured as a receiver (Rx). For practical reasons, the Rx stand is fixed at the same location for the three scenarios. However, the Tx stand is placed at different locations as illustrated in Figure 3a–c. During the measurements, the Tx is configured to transmit one packet every 25 ms. On the Rx side, each received packet includes the RSSI indicating the signal strength of the last received packet, and the packet number for packet loss calculation. Moreover, for each scenario, measurements are performed in two different phases. In the first phase, data are collected using TRs with embedded antenna on both Tx and Rx stands. However, in the second phase, TRs with external antenna are used. Additionally, to reduce the multipath effects and other variables, five measurements (runs) at one minute each are performed at each Tx location. Table 2 summarizes the basic parameters and configurations used in the setup.

## 5. Results, Analysis and Discussion

**Figure 5:**
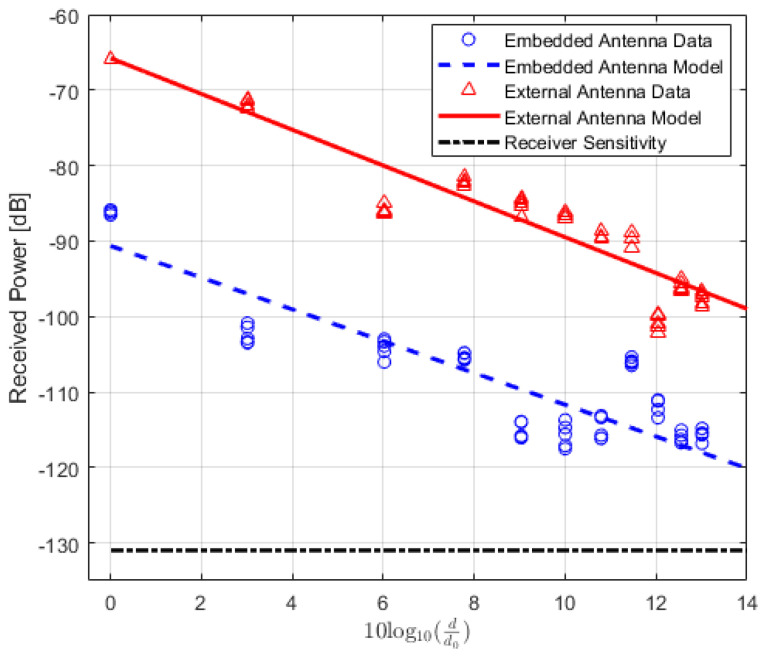
IQRF received power model for Line-of-Sight scenario.

**Figure 6:**
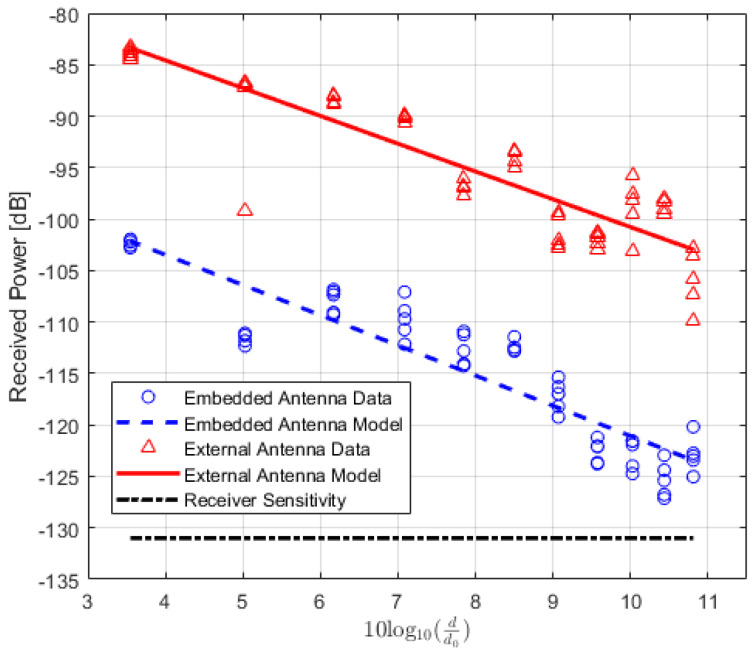
IQRF received power model for Non-Line-of-Sight scenario (one-turn).

**Figure 7:**
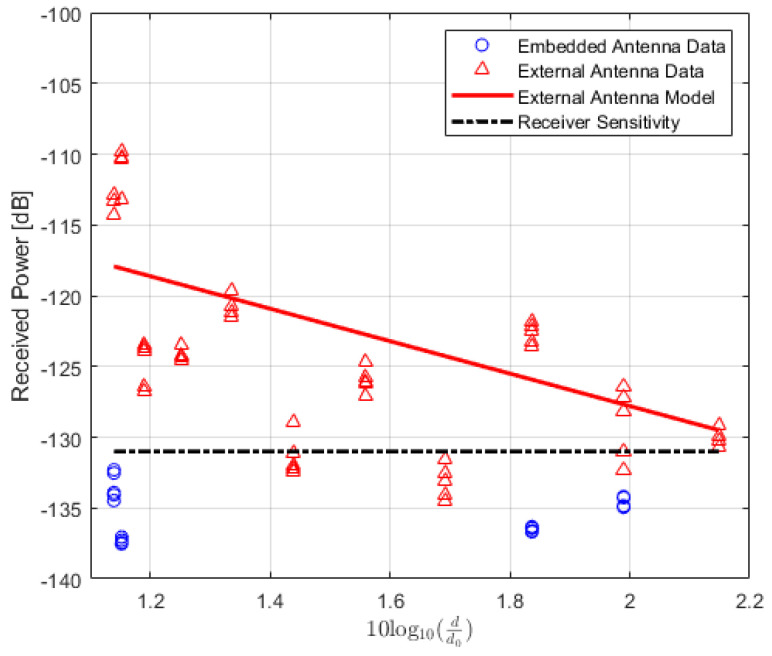
IQRF received power model for Non-Line-of-Sight scenario (two-turns).

The values of the received power or RSSI are recorded and their averages are calculated. In addition, the Packet Delivery Ratio (PDR) is also calculated to analyze the results. PDR is a well-known performance metric that is mostly used in order to analyze the wireless or IoT network performance [42,43]. It provides the relation between the total numbers of packets which are successfully delivered and the total numbers of transmitted packets. For the received power values of the locations where the total number of successfully received packets is lower than 90%, the data are not included in the path loss calculation. The averaged received power values at each location of TRs using either embedded or external antenna with the corresponding PDR values for LoS, NLoS one-turn and NLoS two-turns links are given in Table 3, Table 4 and Table 5, respectively. Corresponding plots with data fitting models are shown in Figure 5, Figure 6 and Figure 7, respectively.

On the other hand, the path loss $PL_{\mathrm{LG}}{\left(d\right)}_{\left[dB\right]}$ of (2) is calculated by considering the gains of the antennas. The following equation is used to calculate the final path loss model for both types of TRs [44].

(21) $$PL_{\mathrm{LG}}{\left(d\right)}_{\left[dB\right]}=P_{\mathrm{Tx}}+G_{\mathrm{Tx}}+G_{\mathrm{Rx}}-P_{\mathrm{Rx}}\left(d\right).$$

**Figure 8:**
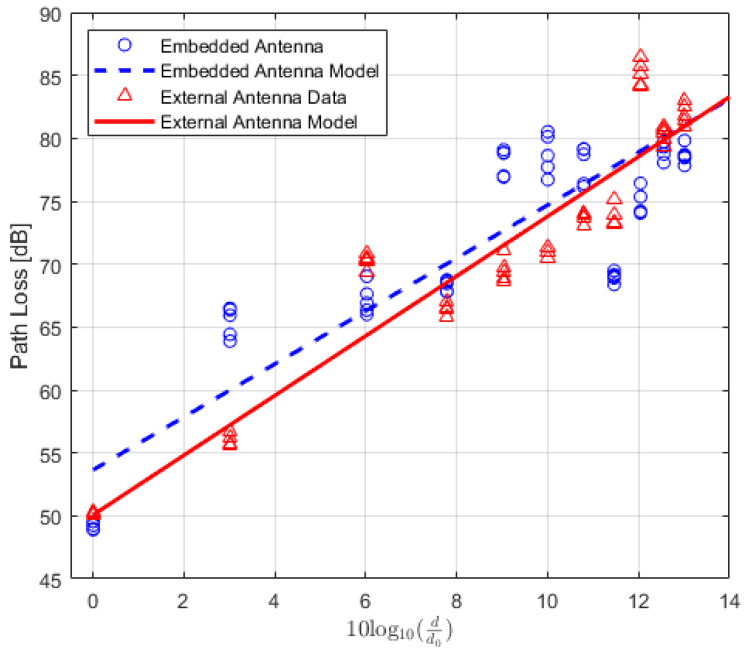
IQRF path loss model for Line-of-Sight scenario.

**Figure 9:**
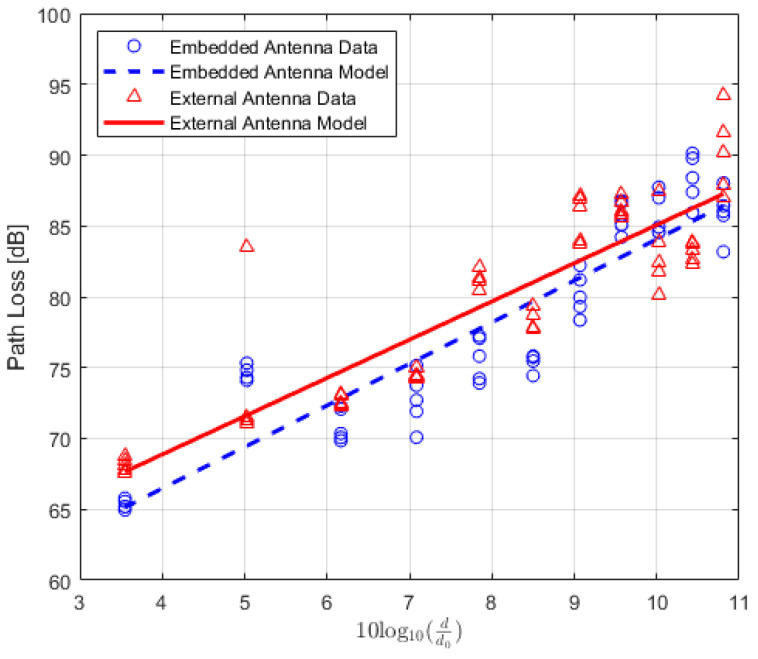
IQRF path loss model for Non-Line-of-Sight scenario (one-turn).

**Figure 10:**
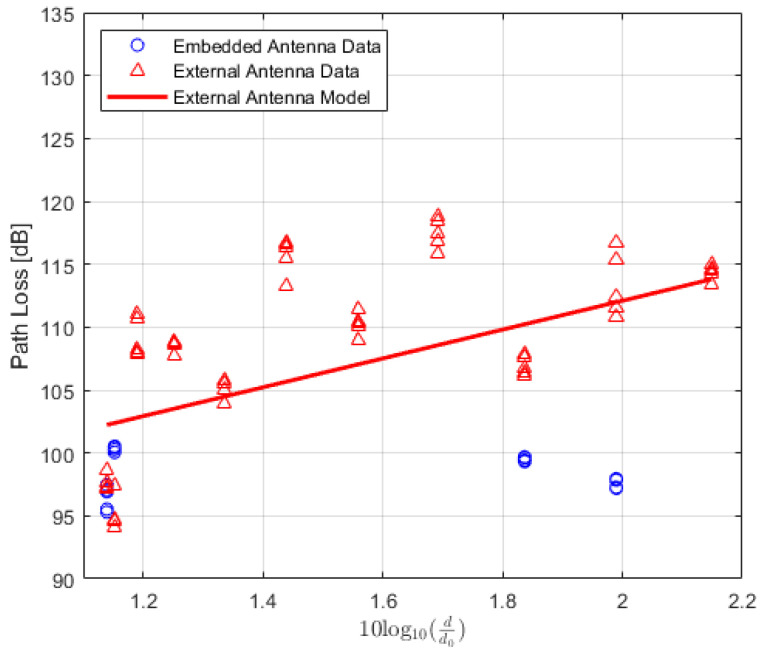
IQRF path loss model for Non-Line-of-Sight scenario (two-turns).

All quantities are expressed in dB, where, $P_{\mathrm{Tx}}$ and $P_{\mathrm{Rx}}$ are the transmitted and received power, respectively. The $G_{\mathrm{Tx}}$ and $G_{\mathrm{Rx}}$ are the transmitter and receiver gains, respectively. Using (2) and (21), the path loss is calculated for both types of TRs and the data are modelled using polynomial curve fitting (in a least-squares sense). The average values of the calculated path loss models along with the corresponding PDR values for LoS, NLoS one-turn and NLoS two-turns links are presented in Table 6, Table 7 and Table 8, respectively. The corresponding plots for the path loss models are shown in Figure 8, Figure 9 and Figure 10. Moreover, path-loss linear regression parameters such as the path loss exponent *n*, the losses $L(d_{0})$ at a far-field reference distance $d_{0}$, the correlation factor between measured and predicted values represented by $R^{2}$, the standard deviation σ, and finally the estimated range for LoS, NLoS one-turn and NLoS two-turns links are presented in Table 9, Table 10 and Table 11, respectively.

**Figure 11:**
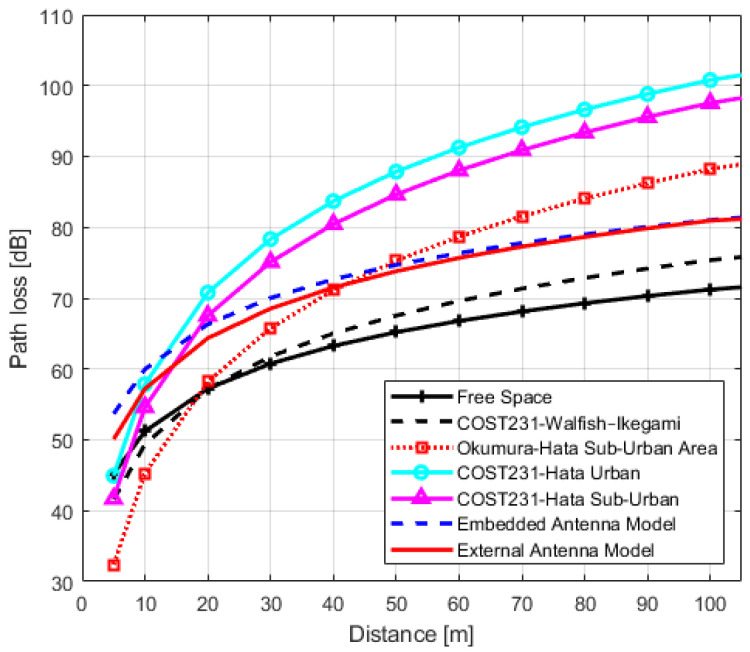
IQRF path loss model comparison with other models for Line-of-Sight scenario.

**Figure 12:**
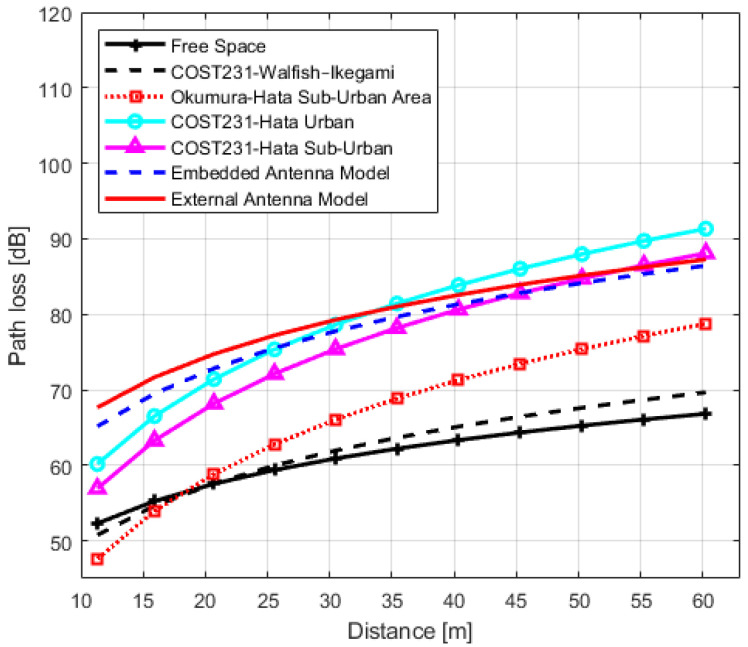
IQRF path loss model comparison with other models for Non-Line-of-Sight scenario (one-turn).

**Figure 13:**
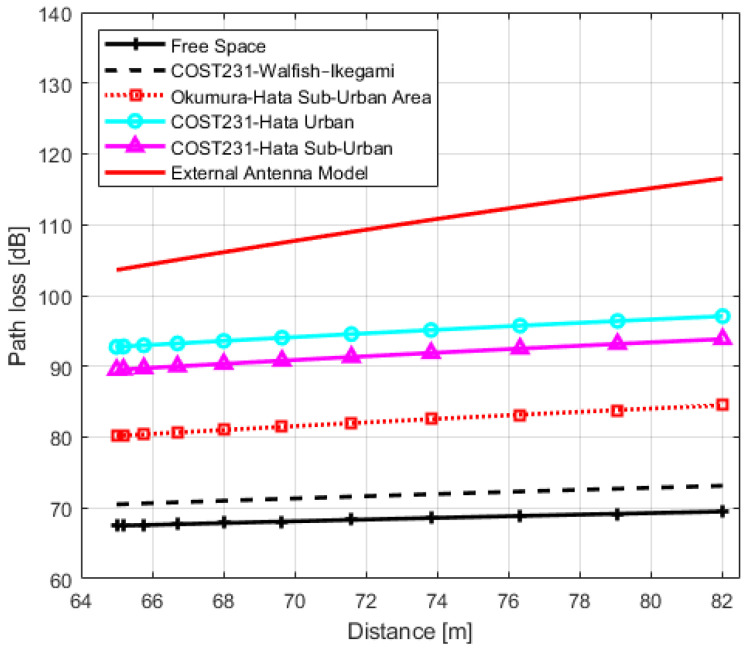
IQRF path loss model comparison with other models for Non-Line-of-Sight scenario (two-turns).

In order to analyze the accuracy of the obtained models and their usability in design, planning and management of IQRF wireless networks in urban environments, we compared IQRF models with the models of Section 3. The graphs of IQRF path loss models for LoS, NLoS one-turn and NLoS two-turns links with models of Section 3 are shown in Figure 11, Figure 12 and Figure 13, respectively. The paramters used for plotting the obtained models are presented in Table 6, Table 7 and Table 8, respectively. In order to compare these models with the models of Section 3, the Root Mean Square Errors (RMSE) method is employed. The comparison results are presented in Table 12.

For IQRF TRs with embedded antenna in the LoS scenario, the COST231 Walfisch–Ikegami model has better prediction accuracy with a minimal error of 9.00 dB when compared to other models. On the other hand, both the Okumura–Hata Suburban and Free-space models are also considered models with minimal errors of 10.33 dB and 10.44 dB, respectively. However, by comparing the models’ exponents, it can be seen from Figure 11 that there is an important difference in the slope between the Okumura–Hata Suburban and IQRF model. Although the error is relatively minimal, the difference in slope with the Okumura–Hata Suburban model results in a larger deviation at lower and higher distances (locations). As a result, this model becomes inapplicable as an IQRF model for these type of scenarios.

Moreover, the exponent of the obtained IQRF model for TRs using embedded antenna in the same scenario is n=2.10, as presented in Table 9. This value is closer to the free space model’s exponent. In addition, the intercept term difference is approximately 10 dB. The same discussion can be applied for COST231 Walfisch–Ikegami but reservedly with the slope value of this model. Hence, for IQRF network implementation in an urban environment using TRs with embedded antenna in an LoS link, Free-space and COST231 Walfisch–Ikegami (with bias to Free-space) models plus an intercept of 10 dB can be used.

For IQRF TRs with external antenna in the same scenario, the minimal errors of 8.03 dB, 8.10 dB and 10.16 dB are also obtained for the Okumura–Hata Suburban, COST231 Walfisch–Ikegami and Free-space models, respectively. Although the error is minimal for the Okumura–Hata Suburban model, there is a difference in slope of this model compared to the IQRF model resulting in the same important deviation at lower and higher distances. For similar reasons, this renders it inapplicable as an IQRF model. On the other hand, the exponent of the obtained IQRF model for these types of TRs is n=2.37. In this case, it is closer to the COST231 Walfisch–Ikegami and Free-space exponents with an intercept term difference of approximately 10 dB, making these two models (with bias to COST231 Walfisch–Ikegami model) more efficient.

In the NLoS one-turn scenario, the best matches for TRs with embedded antenna can both be COST231-Hata for urban and suburban models with error values of 4.38 dB and 4.76 dB, respectively. This is also the case for TR with an external antenna with error values 4.72 dB and 5.94 dB, respectively. Finally, in the NLoS two-turns scenario, for a transceiver with an external antenna, the closest model that can be considered with a minimal error of 15.36 dB, is the COST231-Hata Model for urban areas, but reservedly since the goodness of fit measure $R^{2}$ for this case is relatively low, and the log-distance model is likely an unsuitable model in these types of scenarios.

**Figure 14:**
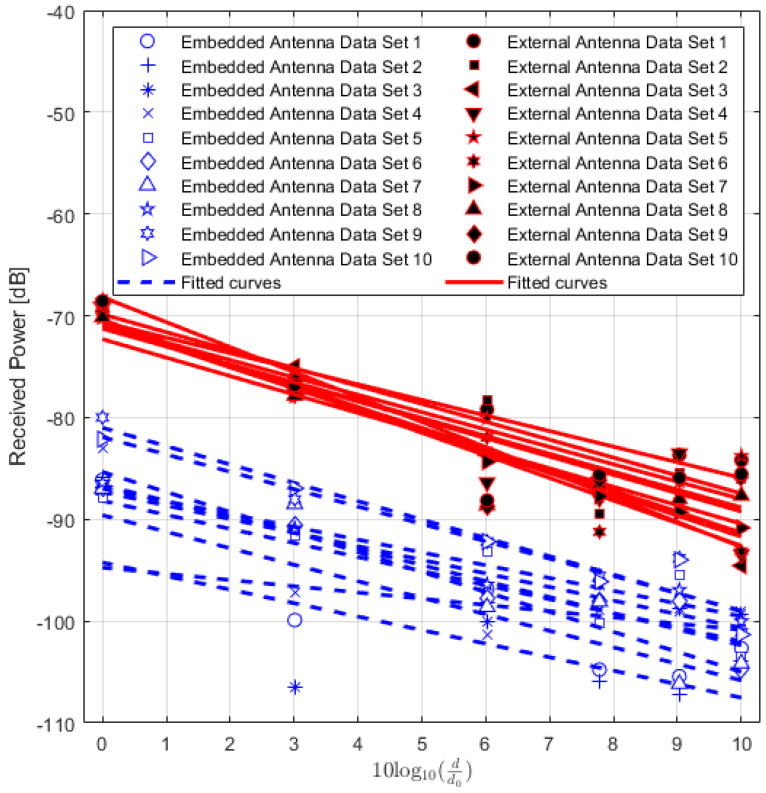
IQRF received power variation comparison for both transceivers with embedded and external antennas.

Furthermore, the performance of both types of IQRF TRs in terms of received power are compared. In this case, ten sets of measurements of the RSSI in an LoS environment in an open field (stadium) are conducted. Each set of data is comprises six measurements at six different locations 5, 10, 20, 30, 40 and 50 m from the fixed Rx stand. In order to keep the Tx stand at the same place, the locations are marked on the ground. Each set of data is modelled with one fitting curve. The results are shown in Figure 14.

From the figure, it can be seen that the results show a larger spread of values for TRs with the embedded antenna (MLA) compared to TRs with the external antenna (SLDA). For TRs with embedded antenna, the spread is minimum, and the maximum average difference is about 15 dB. However, for TRs with external antenna, the minimum and maximum average differences are about 7 dB. Although similar measurements are conducted for both types of transceivers, TRs with embedded antenna are inconsistent. SLDA antennas are usually more efficient as the imaginary component of its complex input impedance is zero [45]. Thus, this represents an important result for the practical use of IQRF. TRs with the embedded antenna seems to be much more sensitive to fading effects, possibly due to the coupling between the antenna and the nearby structure of the circuit board with the electronics and the casing. In addition, the average of its received power level is lower compared to that for the TRs with external antenna. The reason can be linked to the small embedded antenna gain (−8.5 dBi) [29] causing the stochastic noise to exhibit a relatively larger effect. In other words, these results show that the use of IQRF TRs with an external antenna provides better and stable received power and less sensitivity to background noise when compared with TRs with embedded antenna. Hence, a stable IQRF network with longer ranges between nodes could be achieved.

Overall, it is worth mentioning that by using the obtained received power fitting models, the maximum range for IQRF TRs with embedded antenna in an urban environment in the LoS scenario is estimated to be 420 m as listed in Table 9. As discussed in [9] the theoretical range for these types of TRs is 1 km in an LoS setup assuming that there are no obstacles between Tx and Rx by using some sort of range extender. However, if TRs are used without the range extender, then the range shrinks in half (500 m) as mentioned in the IQRF technical documents [29]. The comparison shows an 80-meter difference between the theoretical (technical) and the practical data. This is due to the fact that the TRs are installed in a more dense environment (urban). Additionally, in the NLoS one-turn scenario, the maximum range for these types of TRs is estimated to be 110 m. However, for the NLoS two-turns scenario, the maximum range is not presented as the models could not be calculated. This can give an indication about the range limitations of IQRF TRs (network) with embedded antennas in similar scenarios. On the other side, for TRs with an external antenna, the maximum range is larger. In the LoS scenario, it is estimated to be 2800 m. In this case, there are no technical data available about the maximum range using these types of TRs. Hence, the obtained range is not compared as in the case of TRs with an embedded antenna. Furthermore, in the NLoS one-turn scenario, the range is 660 m. Finally, for the NLoS two-turns scenario, the maximum range is around 90 m. Beyond these ranges, packets may start becoming lost.

As a summary, Table 13 lists the achieved results from this study.

## 6. Conclusions and Future Work

In this paper, empirical path loss models of two types of IQRF transceivers are calculated through the measurement of received power in three different scenarios, LoS, NLoS one-turn and NLoS two-turns. IQRF obtained empirical path loss models were compared with other well-known models to analyze the accuracy of the achieved empirical IQRF models. To this end, the RMSE between the obtained IQRF models and other models was calculated. The results show that in an LoS environment the models that agree well with the IQRF model employing transceivers with embedded and external antennas are the free-space and COST231 Walfisch–Ikegami models, respectively, with an offset of approximately 10 dB. Moreover, for the NLoS one-turn environment, IQRF models for both types of transceivers agree well with two models: COST231-Hata for urban areas, and COST231-Hata for suburban areas. In the NLoS two-turns environment, for a transceiver with an external antenna, the closest model with minimal error is the COST231-Hata Model for urban areas. Furthermore, an IQRF transceiver with embedded antenna was shown to receive power below that of those with external antenna by approximately 20 dB, and was also shown to be more sensitive to fading effects, showing a fluctuation of about 10 dB more than transceivers with external antennas. Finally, maximum ranges of IQRF transceivers are also estimated.

The PDR values for locations (distances) in three scenarios are calculated. From the results, it is observed that the PDR changes inconsistently when increasing the distance. This comes from the fact that the reflected signals are constructed differently in different Tx placements (locations). Hence, longer ranges are required to observe the variation pattern of the PDR; however, this is outside the scope of this study and can be a part of future work.

Another important conclusion is that although IQRF technology can be an alternative to short- and long-range IoT technologies, it is clear that IQRF has a better coverage compared to short-range technologies. However, when compared with long-range technologies, the same range could be achieved using the multi-hops scheme. Nevertheless, to achieve more robust and reliable results regarding coverage in outdoor environments, another experimental study is planned to be implemented in the near future.

## Figures and Tables

**Table 1:** Comparison between the empirical path loss models for outdoor environments.

Model	Frequency Range (MHz)	Pros	Cons
Free-Space	-	Provides the lower bound estimation of link losses.	Applicable for LoS cases.
Log-Distance	-	Easy to implement.	Free space reference distance and path loss exponent need to be appropriately selected or obtained for the propagation environment.
Okumura–Hata	150–1500	Good in urban areas.	Slow response to rapid changes in the propagation environment, the base station or transmitter antenna needs to be higher than the receiver or mobile antenna height.
COST231-Hata	800–2000	Computationally simple.	Its application is limited to large and small macro-cells. It is not appropriate for micro-cell coverage area prediction.
COST231 Walfisch–Ikegami	800–2000	Efficient when the distance between the transmitter and the receiver is large.	Computationally complex, and highly affected by urban structures and geometries.

**Table 2:** Measurement setup parameters.

Parameter	Value	Unit
Transmission Frequency	868.35	MHz
Tx power	10	dBm
Embedded antenna gain	−8.5	dBi
External antenna gain	2.15	dBi
Tx, Rx antennas height above the terrain	2	m
Packets rate	40	Packet/s
Packet size	3	Bytes
Measurement duration	60	s
Number of measurements for each location	5	-

**Table 3:** Measured average of received power and Packet Delivery Ratio for Line-of-Sight scenario.

Antenna Type	Parameter	$10\log_{10}\left(\frac{d}{d_{0}}\right)$, $d_{0}=5$ [m]										
		**0.00**	**3.01**	**6.02**	**7.78**	**9.03**	**10.00**	**10.79**	**11.46**	**12.04**	**12.55**	**13.01**
Embedded	RSSI [dB]	−86.13	−102.42	−104.18	−105.30	−115.14	−115.74	−114.91	−105.99	−111.84	−116.11	−115.68
	PDR [%]	97.17	97.10	97.09	97.09	96.90	96.66	96.84	97.22	97.53	97.46	97.33
External	RSSI [dB]	−65.89	−71.67	−85.91	−82.17	−85.24	−86.64	−89.37	−89.43	−100.83	−95.96	−97.62
	PDR [%]	97.59	97.65	97.73	97.61	97.49	97.61	97.81	97.83	97.72	97.67	97.65

**Table 4:** Measured average of received power and Packet Delivery Ratio for Non Line-of-Sight scenario (one turn).

Antenna Type	Parameter	$10\log_{10}\left(\frac{d}{d_{0}}\right)$, $d_{0}=5$ [m]										
		**3.55**	**5.03**	**6.17**	**7.09**	**7.85**	**8.50**	**9.07**	**9.57**	**10.02**	**10.43**	**10.81**
Embedded	RSSI [dB]	−102.34	−111.67	−107.93	−109.72	−112.66	−112.45	−117.21	−122.56	−122.75	−125.33	−122.88
	PDR [%]	97.37	97.27	97.38	97.25	97.21	97.39	96.95	95.20	95.41	90.33	96.26
External	RSSI [dB]	−83.85	−89.41	−88.33	−90.18	−96.75	−93.96	−101.31	−101.99	−98.82	−98.88	−105.90
	PDR [%]	97.90	96.68	96.88	96.77	97.10	96.79	96.68	96.77	96.93	96.95	96.34

**Table 5:** Measured average of received power and Packet Delivery Ratio for Non-Line-of-Sight scenario (two turns).

Antenna Type	Parameter	$10\log_{10}\left(\frac{d}{d_{0}}\right)$, $d_{0}=50$ [m]										
		**1.14**	**1.15**	**1.19**	**1.25**	**1.34**	**1.44**	**1.56**	**1.69**	**1.84**	**1.99**	**2.15**
Embedded	RSSI [dB]	−133.46	−137.27	-	-	-	-	-	-	−136.49	−134.63	-
	PDR [%]	89.09	44.90	-	-	-	-	-	-	61.38	95.88	-
External	RSSI [dB]	−113.26	−110.77	−124.84	−124.21	−120.89	−131.35	−125.96	−133.16	−122.66	−129.05	−130.05
	PDR [%]	94.88	97.82	94.13	95.62	96.84	80.81	96.06	62.98	96.79	79.27	78.99

**Table 6:** Measured average path loss and Packet Delivery Ratio for Line-of-Sight scenario.

Antenna Type	Parameter	$10\log_{10}\left(\frac{d}{d_{0}}\right)$, $d_{0}=5$ [m]										
		**0.00**	**3.01**	**6.02**	**7.78**	**9.03**	**10.00**	**10.79**	**11.46**	**12.04**	**12.55**	**13.01**
Embedded	PL [dB]	49.13	65.42	67.18	68.30	78.14	78.74	77.91	68.99	74.84	79.11	78.68
	PDR [%]	97.17	97.10	97.09	97.09	96.90	96.66	96.84	97.22	97.53	97.46	97.33
External	PL [dB]	50.19	55.97	70.21	66.47	69.54	70.94	73.67	73.73	85.13	80.26	81.92
	PDR [%]	97.59	97.65	97.73	97.61	97.49	97.61	97.81	97.83	97.72	97.67	97.65
Free-Space	PL [dB]	45.20	51.22	57.24	60.76	63.26	65.20	66.78	68.12	68.72	70.30	71.22
COST231 W-I	PL [dB]	41.54	49.37	57.20	61.78	65.02	67.54	69.60	71.34	72.12	74.18	75.37
O-H SubUrban	PL [dB]	32.35	45.28	58.20	65.76	71.12	75.28	78.68	81.55	82.84	86.24	88.20
COST231 Hata Urban	PL [dB]	44.93	57.85	70.78	78.34	83.70	87.86	91.26	94.13	95.42	98.82	100.78
COST231 Hata SubUrban	PL [dB]	41.70	54.62	67.54	75.10	80.46	84.62	88.02	90.90	92.18	95.58	97.55

**Table 7:** Measured average path loss and Packet Delivery Ratio for Non-Line-of-Sight scenario (one turn).

Antenna Type	Parameter	$10\log_{10}\left(\frac{d}{d_{0}}\right)$, $d_{0}=5$ [m]										
		**3.55**	**5.03**	**6.17**	**7.09**	**7.85**	**8.50**	**9.07**	**9.57**	**10.02**	**10.43**	**10.81**
Embedded	PL [dB]	65.34	74.67	70.93	72.72	75.66	75.45	80.21	85.56	85.75	88.33	85.88
	PDR [%]	97.37	97.27	97.38	97.25	97.21	97.39	96.95	95.20	95.41	90.33	96.26
External	PL [dB]	68.15	73.71	72.63	74.48	81.05	78.26	85.61	86.29	83.12	83.18	90.20
	PDR [%]	97.90	96.68	96.88	96.77	97.10	96.79	96.68	96.77	96.93	96.95	96.34
Free-Space	PL [dB]	52.29	55.25	57.53	59.37	60.89	62.20	63.33	64.34	65.25	66.07	66.81
COST231 W-I	PL [dB]	50.77	54.61	57.58	59.97	61.95	63.64	65.12	66.43	67.61	68.67	69.65
O-H SubUrban	PL [dB]	47.58	53.93	58.83	62.77	66.04	68.84	71.28	73.45	75.39	77.14	78.75
COST231 Hata Urban	PL [dB]	60.16	66.51	71.41	75.35	78.62	81.42	83.86	86.02	87.96	89.72	91.33
COST231 Hata SubUrban	PL [dB]	56.93	63.27	68.17	72.11	75.39	78.19	80.63	82.79	84.73	86.49	88.10

**Table 8:** Measured average path loss and Packet Delivery Ratio for Non-Line-of-Sight scenario (two turns).

Antenna Type	Parameter	$10\log_{10}\left(\frac{d}{d_{0}}\right)$, $d_{0}=5$ [m]										
		**1.14**	**1.15**	**1.19**	**1.25**	**1.34**	**1.44**	**1.56**	**1.69**	**1.84**	**1.99**	**2.15**
Embedded	PL [dB]	96.46	100.27	-	-	-	-	-	-	99.49	97.63	-
	PDR [%]	89.09	44.90	-	-	-	-	-	-	61.38	95.88	-
External	PL [dB]	97.56	95.07	109.14	108.51	105.19	115.65	110.26	117.46	106.96	113.35	114.35
	PDR [%]	94.88	97.82	94.13	95.62	96.84	80.81	96.06	62.98	65.00	79.27	78.99
Free-Space	PL [dB]	67.48	67.50	67.58	67.70	67.87	68.08	68.32	68.58	68.87	69.18	69.50
COST231 W-I	PL [dB]	70.51	70.54	70.64	70.80	71.02	71.29	71.60	71.94	72.32	72.72	73.13
O-H SubUrban	PL [dB]	80.17	80.23	80.39	80.66	81.02	81.46	81.97	82.55	83.17	83.82	84.51
COST231 Hata Urban	PL [dB]	92.75	92.81	92.97	93.23	93.59	94.04	94.55	95.12	95.74	96.40	97.08
COST231 Hata SubUrban	PL [dB]	89.52	89.57	89.73	90.00	90.36	90.80	91.32	91.89	92.51	93.17	93.85

**Table 9:** Path loss regression coefficients and statistical results for Line-of-Sight scenario.

Antenna Type	*n*	$L(d_{0})$ [dB]	$R^{2}$	σ [dB]	$d_{\max}$ [m]
Embedded	2.10	53.65	0.80	4.54	420
External	2.37	50.08	0.93	3.16	2800

**Table 10:** Path loss regression coefficients and statistical results for Non-Line-of-Sight scenario (one turn).

Antenna Type	*n*	$L(d_{0})$ [dB]	$R^{2}$	σ [dB]	$d_{\max}$ [m]
Embedded	2.93	54.74	0.83	3.03	110
External	2.70	58.07	0.79	3.14	660

**Table 11:** Path loss regression coefficients and statistical results for Non-Line-of-Sight scenario (two turns).

Antenna Type	*n*	$L(d_{0})$ [dB]	$R^{2}$	σ [dB]	$d_{\max}$ [m]
Embedded	-	-	-	-	-
External	11.47	89.16	0.24	5.03	90

**Table 12:** Comparison of IQRF models with empirical models of Section 3.

	RMSE for LoS [dB]		RMSE for NLoS one turn [dB]		RMSE for NLoS two turns [dB]	
Path Loss Models	TR Antenna Type		TR Antenna Type		TR Antenna Type	
	Embedded	External	Embedded	External	Embedded	External
Free-Space	10.44	10.16	17.40	18.80	-	40.78
COST231 W-I	9.00	8.10	16.16	17.61	-	37.54
O-H SubUrban	10.33	8.03	12.26	13.79	-	27.37
COST231 Hata Urban	15.44	14.33	4.38	4.72	-	15.36
COST231 Hata SubUrban	13.20	11.78	4.76	5.94	-	18.38

**Table 13:** Summary of the achieved results.

Measurement Scenario	The Performance of the Models	The Effects of Antennas on the Performance of IQRF Transceivers
LoS Link	COST231-Walfisch–Ikegami and Free-Space model can be used to predict path loss. Okumura–Hata model is inapplicable in path loss prediction.	Transceivers with embedded antenna are more sensitive to fading effects. The maximum range for the transceiver with embedded antenna is estimated to be 420 m. The maximum range for the transceiver with external antenna is estimated to be 2800 m.
NLoS: One-Turn Link	COST231-Hata for urban and suburban models can be used to predict path loss. Log-Distance and Okumura–Hata model are inapplicable in path loss prediction.	Transceivers with an external antenna give better and stable results. The maximum range for the transceiver with embedded antenna is estimated to be 110 m. The maximum range for the transceiver with external antenna is estimated to be 660 m.
NLoS: Two-Turns Link	COST231-Hata for the urban model has better prediction accuracy. Log-Distance and Okumura–Hata model are inapplicable in path loss prediction.	The use of transceivers with embedded antenna is inapplicable. The maximum range for the transceiver with external antenna is estimated to be 90 m.

## Data Availability

The data presented in this study are available on request from the corresponding author.
